# Relationship between tumor size and disease stage in non-small cell lung cancer

**DOI:** 10.1186/1471-2407-10-474

**Published:** 2010-09-02

**Authors:** Fu Yang, Haiquan Chen, Jiaqing Xiang, Yawei Zhang, Jianhua Zhou, Hong Hu, Jie Zhang, Xiaoyang Luo

**Affiliations:** 1 Department of Thoracic Surgery, Fudan University Shanghai Cancer Center, 270 Dong'an Road, Shanghai 200032, China; 2 Department of Oncology, Shanghai Medical College, Fudan University, 270 Dong'an Road, Shanghai 200032, China

## Abstract

**Background:**

Whether tumor size and stage distribution are correlated remains controversial. The objective is to assess the relationship between tumor size and disease stage distribution in non-small cell lung cancer (NSCLC).

**Methods:**

We conducted a retrospective analysis of 917 cases of NSCLC that were resected in the Cancer Hospital of Fudan University and Shanghai Sixth Hospital between January 2000 and February 2009. Tumor sizes were grouped into five categories: ≤20 mm, 21 to 30 mm, 31 to 50 mm, 51 to 70 mm and ≥71 mm.

**Results:**

Age and tumor size affected stage distribution: patients 60 years or older had a higher percentage of N0M0 disease than patients younger than 60 years (61.67% vs. 44.85%, p < 0.01). The smaller the tumor, the more likely the disease was N0M0 status (p < 0.05). For tumors ≤20 mm in diameter, the proportion of cases with N0M0 status was 70.79%, compared to 58.88% for 21 to 30 mm, 48.03% for 31 to 50 mm, 47.55% for 51 to 70 mm, 33.33% for ≥71 mm. The mean (± SD) tumor size of cases with N0M0 status was 37.17 ± 21.34 mm, compared to 45.75 ± 23.19 mm for cases with other status.

**Conclusions:**

There is a statistically significant relationship between tumor size and distribution of disease stage of primary NSCLC tumors: the smaller the tumor, the more likely the disease is N0M0 status.

## Background

Lung cancer is the leading cause of cancer-related death worldwide. A total of 497,908 new lung cancer cases and 428,938 deaths from lung cancer are estimated to have occurred in China in 2005 [1,2]. When diagnosis and treatment are initiated early, lung cancer can be cured. In the database of IASLC, the 5-year survival rate of patients with pathologic and clinical stage IA non-small cell lung cancer (NSCLC) is 73% and 50%, respectively [3]. In contrast, the 5-year survival is only 13% and 2% for patients with pathologic and clinical stage IV disease, respectively [3]. Unfortunately, 75%~80% patients with NSCLC are diagnosed with locally advanced or advanced disease [4], resulting in a dismal overall 5-year survival of 15.7% [5,6].

This state implies that to improve survival, efforts should be made to detect NSCLC in earlier stages so that curative surgery can be performed. Lung cancer screening trials with conventional chest radiography have failed to decrease lung cancer mortality [7-9]. However, the use of low-dose helical computed tomography (CT) scans has refueled the impetus to detect lung cancer at earlier stages. Compared to chest tomography, CT scans can detect non-calcified nodules three times more commonly and malignant nodules four times more often [10-12]. Advocates of low-dose CT screening believe that the smaller the lesion, the more likely it is to be in an early stage. Tumor size is an important characteristic of the T descriptors in the seventh edition of the TNM classification for lung cancer [3], but whether tumor size correlates with stage distribution in NSCLC remains controversial. This study was conducted to investigate the relationship between tumor size and stage distribution in nearly 1000 Chinese patients with resected NSCLC.

## Methods

Between January 2000 and February 2009, 1083 patients underwent surgery with curative intent for primary NSCLC in the Cancer Hospital of Fudan University and the Shanghai Sixth Hospital. Inclusion criteria included complete pathologic staging according to the TNM classification system of the UICC/AJCC and adequate documentation of tumor size, the type of lymphadenectomy was systematic nodal dissection. Cases were retrospectively analyzed, and all data were obtained from the medical records, including gender, age, tumor location (right or left side, upper or lower or middle lobe), histology, tumor size, pathologic stage and the presence or absence of lymph node or distant metastases. Routine pre-operative examination included chest CT, abdominal CT or ultrasonography or magnetic resonance imaging, brain CT or magnetic resonance imaging, and bone scanning. The final pathologic stage and the status of lymph node or distant metastases were determined based on pathology reports and clinical data. It was classified as N0 (no metastases), N1 (only ipsilateral peribronchial, hilar, and/or intrapulmonary metastases), N2 (ipsilateral mediastinal and/or subcarinal metastases, no contralateral), or N3 (contralateral mediastinal and/or hilar, scalene, or supraclavicular metastases). Status of distant metastases was classified as M0 (absent) or M1 (present). N0M0 status was defined as no metastases, other status were defined as metastases. Tumor size was defined by greatest diameter based on the pathology report. Histology was divided into five categories according to the 2004 World Health Organization classification of lung tumor: squamous cell carcinomas, adenocarcinomas, adenosquamous cell carcinomas, large cell carcinomas, and other histologic types of primary NSCLC. Totally 158 patients were excluded. 148 patients were excluded because of the type of lymphadenectomy was not systematic nodal dissection. 10 patients were excluded because of the tumor size was not clear from the pathology report. And 8 patients were excluded because of neoadjuvant chemotherapy or chemoradiotherapy. The remaining 917 cases were received lobectomy or pneumonectomy.

According to the revision of the T descriptors in the seventh edition of the TNM classification for lung cancer [13], we classify the 917 remaining cases of NSCLC into the following five tumor size categories according to the greatest diameter of tumor: ≤20 mm, 21~30 mm, 31~50 mm, 51~70 mm, and ≥71 mm. We focused principally on the frequency of N0M0 status in these categories. Tumor size was used as a continuous variable. Both parametric and nonparametric methods were used to compare stage distributions relative to tumor size. Univariate associations between disease stage and gender, age, location of tumor, histology, and tumor size were explored using χ^2 ^tests. The independent effect of tumor size as categorical variables on disease stage was analyzed using a logistic regression model. All the analyses were conducted using the statistical software (SPSS15.0). All P values are two sided and considered statistically significant when less than 0.05.

The study was conducted in accordance with the principles of the Helsinki Declaration. And it was approved by the Ethics Committee of Cancer Hospital of Fudan University. Because this was a retrospective analysis, patient's consent was not required.

## Results

A total of 917 patients (255 women, 27.81%, 662 men, 72.19%) with primary NSCLC met the inclusion criteria and were analyzed. The mean (± standard deviation) age was 60.15 ± 9.80 years, with a range from 20 to 83 years. Of the 917 cases, 339 cases (36.97%) were squamous cell carcinomas, 448 cases (48.85%) adenocarcinomas, 57 cases (6.22%) adenosquamous cell carcinomas, and 35 cases (3.82%) large cell carcinomas. The remaining 38 cases (4.14%) were other histologic types of primary NSCLC. The tumor was left-sided in 418 cases (45.58%), right-sided in 499 cases (54.42%). 522 cases (56.92%) were upper-lobe, 63 cases (6.87%) were middle-lobe, 332 cases (36.21%) were lower-lobe. Among the 917 cases, 178 (19.41%) were ≤20 mm in diameter, 214 (23.34%) cases were 20~30 mm, 304 (33.15%) cases were 30~50 mm, 143 (15.59%) cases were 50~70 mm, and 78 (8.51%) cases were ≥71 mm. The mean number of N1 and N2 nodal stations removed are 2.01 and 4.00, respectively. And the mean number of lymph node removed is 18.18. 492 cases of non-small cell lung cancer were found to be N0M0 status. There were 23 cases of stage IV disease. Among these, 4 cases were found to be distant metastases before operation but the patients and family members demanded surgery treatment. And 19 patients were found to have pleural nodules or malignant pleural dissemination at operation. Three cases of NSCLC were found to be N3 at operation.

Univariate analysis revealed that there were no associations between sex, location of lung cancer, pathology and stage distribution, while age and tumor size affected stage distribution significantly. Patients 60 years or older had higher percentages of N0M0 than the ones younger than 60 years (61.67% vs. 44.85%, p < 0.001). Univariate analysis revealed that tumor size affected stage distribution: the smaller the tumor, the more likely the disease was N0M0. The proportion of cases with no metastasis (N0M0) was 53.65% overall. For tumors ≤20 mm in diameter, the proportion of cases with N0M0 was 70.79% (95% confidence interval (CI), 64.11%~77.47%), compared to 58.88% (95% CI, 52.29%~65.47%) for 21 to 30 mm, 48.03% (95% CI, 42.41%~53.64%) for 31 to 50 mm, 47.55% (95% CI, 39.15%~55.95%) for 51 to 70 mm, 33.33% (95% CI, 22.87%~43.79%) for ≥71 mm (Table 1). The proportions of cases with N0M0 were significantly different between all the successive categories (p < 0.05), except between tumors 31 to 50 mm and 51 to 70 mm (p = 1.00). The proportions of cases with N0M0 were statistically significantly different among all the non-consecutive categories according to tumor size (p < 0.05). Tumor size did affect the distribution of stage in the multivariate analysis with the following variables entered to the multivariate analysis: gender, age, location of tumor, histology, categories of tumor size.

**Table 1:** Distribution of disease stage according to tumor size

	Tumor size (mm)					
		
Stage of disease	≤20	21~30	31~50	51~70	≥71	Total
N0M0	126	126	146	68	26	491
N1M0	13	29	48	24	19	133
N2M0	32	52	103	49	29	266
N0-2M1/N3M0-1	7	7	7	2	4	27
Total (% of N0M0)	178(70.79)	214(58.88)	304(48.03)	143(47.55)	78(33.33)	917(53.65)

The mean (± SD) tumor size of cases with N0M0 status was 37.17 ± 21.34 mm, compared to 45.75 ± 23.19 mm for cases with other status. The tumor size of cases with N0M0 disease was significantly smaller than all the other cases (p < 0.01).

## Discussion

The proportion of patients dying from lung cancer in China rose 30.4% from 2000 to 2005 [1,2]. The incidence rate continues to increase due to the high prevalence of smoking and China's large population. Efforts need to be made to detect lung cancer earlier, so patients can live longer.

Proponents of CT screening for lung cancer believe that highly sensitive CT scans may reduce mortality from lung cancer. CT scans reportedly can detect lung cancers with smaller lesions, leading to a significant stage shift from advanced late stage disease to early, more curable stages. According to the new lung cancer staging system, tumor size is an important characteristic of the T descriptors [3]. While more and more studies support that tumor size is an important survival factor in NSCLC [14-17], the association between tumor size and stage distribution is still controversial.

The current study was conducted to analyze the relationship between clinicopathologic factors and disease stage distribution. This study suggests that patients 60 year or older have higher percentage of N0M0 disease than those younger than 60 year (61.67% vs. 44.85%, p < 0.001). Several factors may explain this finding. First, higher proportion of older peoples seek and receive a health examination, as they pay more attention to their physical status. Consistent with this, many patients were found to have incidental NSCLC when under evaluation for other diseases. Second, younger patients tend to have more aggressive NSCLC [18]. The third reason for this is elderly patients are less likely than young ones to be offered surgical treatment if the preoperative work-up showed a more advanced tumor stage. This observation indicates that we cannot ignore lung cancer screening in younger people.

Studies on the effect of tumor size on disease stage have conflicting results [19-22]. Although recent studies of CT screening found relatively high percentages of stage I NSCLC (70%~87%) [23-25], Swensen found no statistically significant stage shift. He explained that the high percentage of detected stage I lung cancers may be due to a larger number of indolent cancers being detected [26]. To test the presumption that smaller lesions represent earlier stage diseases, Heyneman and his colleagues conducted an analysis including 620 cases who presented with pathologically proven primary NSCLC measuring ≤3 cm [19]. They found no statistically significant differences in distribution of disease stage according to tumor size categories. They asserted that CT screening may not result in a shift from advanced stage to earlier stage distribution. However, cases from the study cohort were collected over a 20 year period and included only 25 cases with tumor sizes <1 cm, while the percentage of stage I cases was very high for all categories of tumor sizes. These factors may lower the power to detect a relationship between tumor size and disease stage. Henschke and colleagues demonstrated an association between tumor size and stage distribution in either symptomatic or asymptomatic patients [20,21]. One study utilizing the Surveillance, Epidemiology, and End Results database including 84152 cases primary NSCLC revealed that tumor size did have an effect on disease stage distribution: the smaller the tumor, the more likely the disease was stage I [20]. In another study performed by the I-ELCAP investigators, Henschke concluded that lymph node status had a strong relationship to tumor diameter for NSCLC in screen-diagnosed lung cancers: the percentage of N0M0 cases was higher in patients with smaller tumors [21]. Flieder and his colleagues demonstrated that NSCLC measuring 21~30 mm was twice as likely to have nodal metastases as carcinomas ≤20 mm [22].

**Figure 1:**
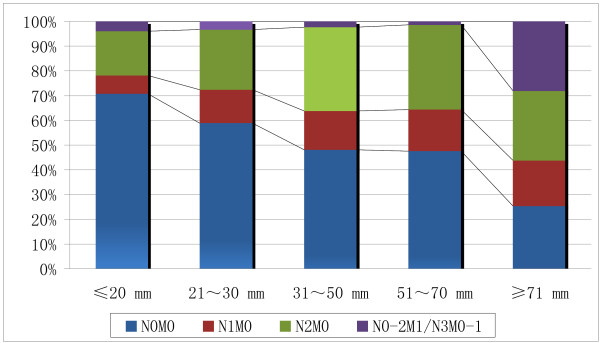
**Proportions of disease stage according to tumor size**. The percentage of N0M0 disease decreased with the increasing of tumor diameter. The proportion of cases with N0M0 was 70.79% for tumor ≤ 20 mm, compared to 58.88% for 21 to 30 mm, 48.03% for 31 to 50 mm, 47.55% for 51 to 70 mm, 33.33% for ≥ 71 mm. The proportions of cases with N0M0 were significantly different between all the successive categories (p < 0.05), except between tumors 31 to 50 mm and 51 to 70 mm (p = 1.00).

Our study suggests that there is a relationship between tumor size and disease stage of NSCLC: the percentage of N0M0 disease decreased with increasing tumor diameter. The proportion of N0M0 disease was 70.79% (95% CI, 64.11%~77.47%) for tumors ≤ 20 mm, compared to 58.88% (95% CI, 52.29%~65.47%) for 21 to 30 mm, 48.03% (95% CI, 42.41%~53.64%) for 30 to 50 mm, 47.55% (95% CI, 39.15%~55.95%) for 51 to 70 mm, 33.33% (95% CI, 22.87%~43.79%) for ≥71 mm. The gradients in the successive percentages of N0M0 were significantly different (p < 0.05), except between tumors 31 to 50 mm and 51 to 70 mm (p = 1.00) (Figure 1). The percentage of N0M0 disease for category of ≤20 mm was significantly higher than all the other categories (p < 0.05), and the gradients in all the non-successive percentages of N0M0 disease were significantly different (p < 0.05). The tumor size of cases with N0M0 was significantly smaller than all the other cases (p < 0.01). Logistic regression model determined the true effect of tumor size on stage distribution.

The current study was different from previous studies [19-21]. In previous studies, thoracic CTs had an accuracy of only 75%~80% in assessing mediastinal lymph nodes in NSCLC patients [27]. Lymph node metastasis was recognized even in clinical T1N0M0 lung cancers smaller than 2 cm [28]. 39% cases showed a mismatch between clinical diameter based on thoracic CT and postsurgical diameter [28]. These factors may lower the accuracy of the results of previous studies. The status of stage and tumor diameter in cases from our study was more accurate than in previous studies, which included cases whose staging and size of lesions were determined only based on clinical examinations. All cases in our study cohort received surgical treatment, and postsurgical pathologic stage and tumor diameter were obtained from pathology reports. However, as in previous studies, our study has a selection bias by including symptomatic and asymptomatic patients. As Henschke said, compared with large tumors, smaller tumors are more likely to be symptomatic due to their lymph node or distant metastases. As smaller tumors are not likely to cause local symptoms and would remain mostly undetected unless they have disseminated, while large cancers are more likely to be symptomatic due to local invasion or compression. This may dilute the size/stage relationship within our database relative to that in a screening database. Our study cohort also included only those who underwent an operation, which could be another source of bias.

The percentage of N2 disease in the 5 categories of our study is higher than the result reported in the IASLC database [29], probably because of a sparse use of invasive staging procedures or PET-CT examination before operation, especially before 2004 in the Cancer Hospital of Fudan University.

The median diameter of lesions missed by chest radiography was 16 mm [30,31], while the median diameter of primary lung cancers detected by CT screening was 15 mm [10-12,24-26]. Sone and colleagues reported that the miss rate of lung cancers ≤ 2 cm on chest radiography was 79% [32]. The mean size of lung cancer lesions detected by chest radiography was 3 cm [33]. In conclusion, our study suggests that tumor size at diagnosis has a definite effect on the stage distribution of NSCLC: smaller lesions represent earlier stage disease. CT screening can detect smaller lesions that represent more early stage lung cancer.

## Conclusions

Tumor size has a definite effect on stage of NSCLC: smaller lesions represent earlier stage disease.

## Abbreviations

CI: confidence interval; CT: computed tomography; NSCLC: non-small cell lung cancer; SD: Standard deviation.

## Competing interests

All the authors declare that they have no actual or potential competing interests including any financial, personal or other relationships with other people or companies/organizations that could inappropriately influence this article.

## Authors' contributions

FY contributed to the design, acquisition of data, analysis of data, and drafting the manuscript. HQC contributed to conception, design, revised the article critically for important intellectual content, and provided final approval of the version to be published. JQX contributed to conception and interpretation of data. YWZ contributed to conception and interpretation of data. JHZ contributed to acquisition of data. HH contributed to acquisition of data. JZ contributed to analysis of data. XYL contributed to acquisition of data. All authors read and approved the final version of the manuscript.

## Pre-publication history

The pre-publication history for this paper can be accessed here:

http://www.biomedcentral.com/1471-2407/10/474/prepub
